# Successful staged repair for a rare type of truncus arteriosus with interruption of the aortic arch and abnormal origin of the left coronary artery

**DOI:** 10.1186/1749-8090-8-136

**Published:** 2013-05-28

**Authors:** Shunji Uchita, Yorikazu Harada, Kentaro Honda, Koji Toguchi, Yoshiharu Nishimura, Tomohiro Suenaga, Takashi Takeuchi, Hiroyuki Suzuki, Yoshitaka Okamura

**Affiliations:** 1 Department of Thoracic and Cardiovascular Surgery, Wakayama Medical University, 811-1 Kimiidera, Wakayama city, Wakayama, 641-8509, Japan; 2 Department of Pediatrics, Wakayama Medical University, Wakayama, Japan; 3 Department of Cardiovascular Surgery, Nagano Children’s Hospital, Nagano, Japan

**Keywords:** Truncus arteriosus, IAA, Abnormal coronary origin, Cat-cry syndrome

## Abstract

We report a successful staged repair for a quite rare combination of truncus arteriosus (TA), Van Praagh type A4, and abnormal origin of the left coronary artery (CA). Furthermore, the case was complicated by a variant of the chromosomal anomaly in cat-cry syndrome. The presence of interruption of the aortic arch (IAA) and abnormal CA origin has been previously reported to increase mortality. To decrease the risk of bronchomalacia in infants, bilateral pulmonary artery banding (PAB) was performed as the first stage procedure for adjusting the pulmonary flow. Staged repair is a useful strategy for infants with complex TA.

## Background

The mortality rate for TA repair is quite high, and the presence of IAA and CA anomaly has been determined as risk factors for death. In addition, the condition of this patient was complicated by a chromosomal anomaly of 46 XX del. (5) (p13.2), which is related to the cat-cry syndrome. Staged surgery was performed to avoid respiratory tract disorder and to decrease the operative risk in this newborn. We report a successful two-stage repair, which consisted of bilateral PAB for an infant who had TA with IAA and abnormal CA origin.

## Case presentation

**Figure 1:**
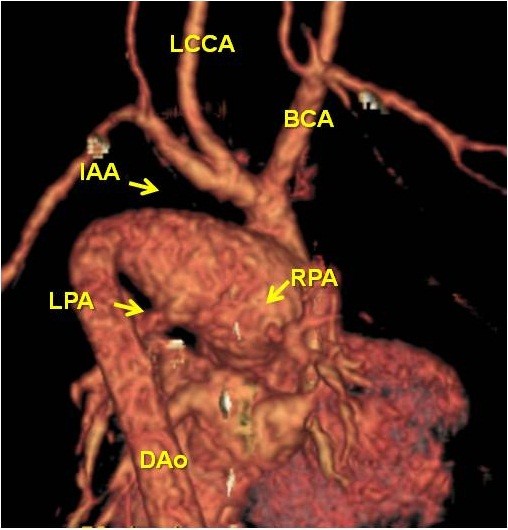
**Posterior 3D CT view.** IAA type A and RPA bifurcated distally to LPA were observed.

**Figure 2:**
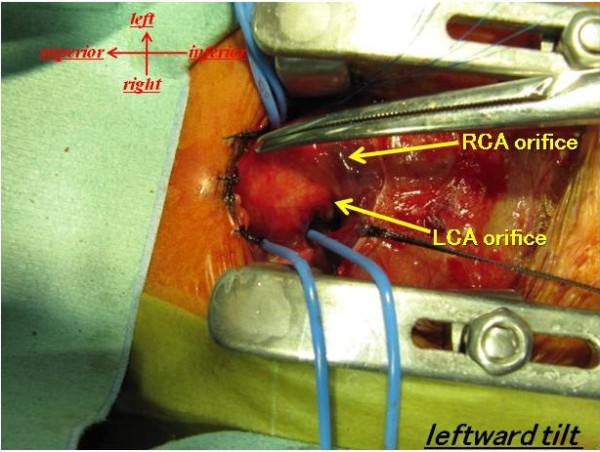
**Surgical view at PAB.** Abnormal origin of LCA arose from the right anterior aspect.

**Figure 3:**
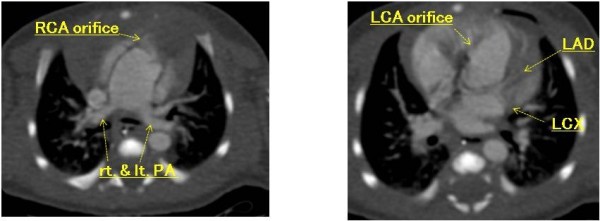
**CT view.** An abnormal LCA originated from the right anterior aspect and extended behind the TA.

**Figure 4:**
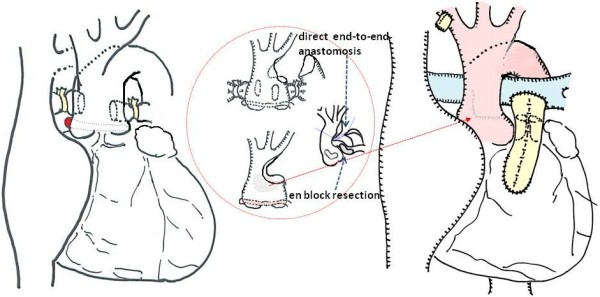
**Operative scheme.** The PA branches were excised and closed by “L” shape. Aortic arch was directly reconstructed. A hand-made bicuspid conduit were applied for RVOT reconstruction. *BCA: brachiocephalic artery, LCCA: left common carotid artery, DAo:descending aorta IAA: interruption of the aorta, LPA: left pulmonary artery, RPA: right pulmonary artery.*

A newborn Japanese girl (gestation period, 37 weeks and 5 days; weight, 2410 g) was delivered by caesarean section. During pregnancy, intrauterine growth retardation and hydramnion were noted. Dyspnea with retraction and labored breathing was observed and intubation was required immediately following birth. TA with IAA type A (Van Praagh type A4) was noted on the echocardiogram and lipoprostaglandin E1 was administered. At 2 days of age, congestive heart failure due to increased pulmonary flow was observed with arterial oxygen saturation (SpO2) of 93% of room air; N2 therapy was started. Computed tomography (CT) scan results showed also Van Praagh type A4 TA with IAA type A and the right pulmonary artery branch bifurcated distally compared to the left pulmonary artery branch (Figure 1). Bilateral PAB was performed through median sternotomy at 7 days of age. A hand-made banding tape with a width of 2 mm, which was obtained from a thin-walled expanded polytetrafluoroethylene (PTFE) graft, was placed around the PA and tightened 8.5 mm in circumference. Abnormal origin of the left CA, which arose from the right anterior aspect, was noted at this time (Figure 2). At the time of review of the preoperative CT images, an abnormal left CA that originated from the right anterior aspect and extended behind the TA was identified (Figure 3). Extubation was performed 1 day after surgery without complications. Percutaneous oxygen saturation was around 80% under room air condition. The result of the chromosomal G-band analysis revealed 46.XX del(5)(p13.2), which is related to the cat-cry syndrome. The truncal valve regurgitation was changed trivial to mild after bilateral PAB by ultrasonic cardiography (UCG). At the age of 1 month, intracardiac repair with aortic arch reconstruction was performed. For aortic arch reconstruction, a wide dissection and mobilization of the descending aorta, TA, PDA, and neck vessels were performed. A cardiopulmonary bypass was established with double arterial (brachiocephalic and thoracic descending aortic) and bicaval direct cannulation with moderate hypothermia. The PDA was ligated and cardiac arrest was induced with blood cardioplegic solution. After the cardiopulmonary bypass, both pulmonary artery branches were snared and a vent was inserted into the left atrium to prevent left ventricular distention. The PA branches were excised en bloc from the truncal vessel and exposed through an oblique incision; the resultant defects were closed primarily by suturing vertically and horizontally (“L” shape). Accurate excision of the pulmonary bifurcation without injury to the truncal valve and positioning of the left CA around just beneath the PA stamp was required. For aortic arch reconstruction, the subaortic arch space had to be considered to avoid compression of the PA branch. A well-mobilized distal arch and descending aorta were directly reconstructed without tension after resection of the ductal tissue. The VSD was closed through right ventriculotomy with a PTFE patch using interrupted pledgeted sutures. After removing the banding tape, a dilator was used to confirm that no stenosis had occurred and that the inner diameter was above the normal value. A hand-made bicuspid conduit with a PTFE tube (12 mm in diameter) and a fan-shaped PTFE sheet (0.1 mm thick) were applied between the right ventricle (RV) and PA to reconstruct the RV outflow tract (Figure 4). Delayed sternum closure was performed 3 days after the surgery. Extubation and weaning from mechanical respiratory support was possible 5 days after sternum closure. Cardiac catheterization at 10 months after total correction showed a well-reconstructed aortic arch without pressure gradient and with mild right pulmonary branch stenosis (30 mmHg) at bifurcation. The tiny truncal valve regurgitation was detected by UCG.

## Discussion

According to Collet and Edwards [1], and Van Praagh [2], TA is usually categorized according to the pattern of the pulmonary origin. The optimal timing and procedure for TA repair were decided on the basis of the morphological characteristics. The presence of IAA, severe truncal valve regurgitation and CA anomalies influence the outcome and mortality [3,4]. On the basis of these risks of complex TA, Adachi et al. [5] investigated combinations of a relationship between the origins of PA and CA in common arterial trunks. The pattern of pulmonary origin and the location of the orifices and relationship to other surrounding structures are very useful and provide important information for surgical correction. In our case, the left CA originated from the right anterior aspect and went around the back of the trunk. The left PA orifice was located at the left posterior aspect and right PA orifice at the right posterior wall close to the ST junction. These patterns were rare and were described as exceptions to their report. Definitive one-stage repair of TA with IAA for low birth weight infants in the newborn period increases the risk of mortality and morbidity, ie, cerebral hemorrhage, reoperation during early childhood associated with small caliber conduit, respiratory distress, and pulmonary branch stenosis caused by a narrow space beneath the aortic arch. In particular, since a genetic abnormality (cat-cry syndrome) was identified in this case, respiratory disorder and respiratory tract infection were concerns after surgery. Hence, in order to avoid some risks induced by cardiopulmonary bypass surgery in the neonatal period, we performed bilateral PAB as the first stage palliative surgery. Bilateral PAB is effective for pulmonary and systemic blood flow balance and for prevention of volume overload. However, ductus patency must be maintained until total correction and pulmonary blood flow might be reduced relatively by growth. For reduced pulmonary flow, Kobayashi et al. [6] reported an adjustable method in which a banding tape was fixed by metal clips and the banding site was dilated by using a balloon catheter. Definitive repair was performed at 1 month after bilateral PAB. To decrease the risk of operative complications, the following operative strategy was considered: the PA was excised en block from the truncal artery to secure autologous continuity; the truncal wall defect was excised and the incision was closed by “L”-shaped suture to avoid left CA injury and truncal valve distortion; the post ductal descending aorta was well mobilized; and sliding end-to-end anastomosis was carried out to reconstruct a smooth aortic arch. RV to pulmonary artery continuity was established with a bicuspid valved conduit, which was passed through the left side of the truncal artery to avoid coronary artery compression.

## Conclusions

We conclude that TA of Van Praagh A4 type is rare and complications due to abnormal CA origin are even rarer. In addition, there is no report of TA with cat-cry syndrome, which represents a risk of respiratory disorder. We report a case of successful two-stage repair of TA with IAA (Van Praagh A4) and chromosomal anomaly of 46.XX del(5)(p13.2).

## Consent

Written informed consent was obtained from the patient for publication of this Case report and any accompanying images. A copy of the written consent is available for review by the Editor-in-Chief of this journal.

## Abbreviations

TA: Truncus arteriosus; CA: Coronary artery; IAA: Interruption of the aortic arch; PTFE: Polytetrafluoroethylene; RV: Right ventricle; PAB: Pulmonary artery banding; UCG: Ultrasonic cardiography

## Competing interests

The authors declare that they have no competing interests.

## Authors’ contributions

SU, YH, KH, KT, YN and YO carried out the surgical procedure, participated in provision of clinical information and reviewed the manuscript. TS, TT and HS carried out the clinical diagnosis and participated in provision of clinical information. All authors read and approved the final manuscript.
